# Supplementary material for: Smooth Interpolating Curves with Local Control and Monotone Alternating Curvature
Source: Comput Graph Forum. 2022 Oct 6;41(5):25–38. doi: 10.1111/cgf.14600 (PMC9827861; doi:10.1111/cgf.14600)
Supplement: Supplementary file 1 — Supplement Material [file CGF-41-25-s001.zip › Local-Smooth-Interpolating-MonoCurvature/extern/clothoids/submodules/quarticRootsFlocke/docs/api-cpp/file_PolynomialRoots-Utils.hh.html]

File PolynomialRoots-Utils.hh — Quartic Roots v1.0.0

### Navigation

- index
- toc
- Quartic Roots »
- File PolynomialRoots-Utils.hh

# File PolynomialRoots-Utils.hh¶

Contents

- Definition (`PolynomialRoots-Utils.hh`)
- Includes
- Included By
- Namespaces
- Typedefs

## Definition (`PolynomialRoots-Utils.hh`)¶

- Program Listing for File PolynomialRoots-Utils.hh

## Includes¶

- `cmath`
- `complex`
- `cstdint`
- `cstdlib`
- `iostream`
- `limits`
- `utility`

## Included By¶

- File PolynomialRoots-Utils.cc

## Namespaces¶

- Namespace PolynomialRoots

## Typedefs¶

- Typedef PolynomialRoots::complexType
- Typedef PolynomialRoots::indexType
- Typedef PolynomialRoots::valueType

### Quick search

### Table of Contents

- Quartic Roots Solver
- C++ API

«
hide menu

menu
sidebar
»

### Navigation

- index
- toc
- Quartic Roots »
- File PolynomialRoots-Utils.hh

© Copyright 2021, Enrico Bertolazzi.
Created using Sphinx 4.2.0.
